# Increased angiogenic factors in the aqueous and vitreous humors after disinsertion of extraocular muscle and the effects of triamcinolone acetate injection

**DOI:** 10.1038/s41598-022-09377-5

**Published:** 2022-03-28

**Authors:** Ha Suk-Gyu, Boram Kang, Jong Suk Song

**Affiliations:** Department of Ophthalmology, Ophthalmology, Korea University College of Medicine, Korea University Guro Hospital, 148, Gurodong-ro, Guro-gu, Seoul, 08308 Korea

**Keywords:** Biomarkers, Medical research, Pathogenesis

## Abstract

The four extraocular rectus muscles in the rabbits were disinserted for induction of anterior segment ischemia (ASI) and the changes in the concentrations of prostaglandin E2 (PGE2), hypoxia-inducible factor-1 (HIF-1α), and vascular endothelial growth factor (VEGF) in the aqueous and vitreous humor were evaluated. Disinsertion of four rectus muscles in rabbits was performed in the right eyes of rabbits (ASI group). The concentrations of PGE2, HIF-1α, and VEGF in the aqueous and vitreous humor were measured at 1, 3, 6, 12, and 24 h by ELISA. The concentrations were compared with those of the fellow eyes (contralateral group) and normal healthy eyes (control group). Subconjunctival injection of triamcinolone acetonide (TA) was administered and three cytokine concentrations in the aqueous humor and vitreous humor were measured at 12 h after TA injection. A total of 48 eyes from 28 rabbits were included. The concentrations of PGE2, HIF-1α, and VEGF in the aqueous humor in the ASI and contralateral groups were significantly higher than those in the control group (p < 0.05, all). The aqueous and vitreous humor concentrations of VEGF in eyes with simultaneous TA injection were significantly lower than were those in the ASI group (p = 0.02, all). The concentration of PGE2, HIF-1α, and VEGF in the aqueous humor was increased after induction of ASI and TA injection seems to be effective in inhibiting VEGF elevation in ASI.

## Introduction

Anterior segment ischemia (ASI) is a potentially serious, but rare complication of ocular surgery. Although the exact prevalence of ASI is unknown, ASI after strabismus surgery occurs in about 1/13,000–1/30,000, according to the first report^1^. ASI is diagnosed on the basis of clinical findings. The clinical features of ASI are variable, ranging from mild iris inflammation to phthisis bulbi with severe visual loss due to decreased blood flow to the anterior segment^2^.

Most cases of ASI after strabismus surgery occur in the early postoperative period. However, there are no reports on the timing of the postoperative occurrence of ASI. In humans, most cases of ASI have been reported after surgical procedures on three or four rectus muscles. The risk of ASI in the four-muscle group was 9.5 times greater than that in the two-muscle group^3^. A dramatic decrease in the blood supply after strabismus surgery to the anterior segment can lead to an increased risk of ASI. The risk factors for ASI also include advanced age, procedures involving multiple muscles, procedures on vertical muscles, hyperviscosity, and systemic vascular diseases^4^.

Angiogenic and inflammatory factors in the eye may contribute in the angiogenesis during acute phase of ASI. Hypoxic damage promotes vessel regeneration by upregulating multiple angiogenic factors, including prostaglandin E2 (PGE2), hypoxia-inducible factor (HIF), and vascular endothelial growth factor (VEGF) in the aqueous humor. A previous study reported that aqueous VEGF could be used as an indicator for the severity of ASI^5^. Another study reported that a blood–aqueous barrier breakdown was observed after tenotomy of the extraocular muscle (EOM) in an animal model^6^. To prevent ischemia, triamcinolone acetonide (TA) has been used to treat various ocular inflammation, edema, and ischemic diseases^7^.

To the best of our knowledge, there is no objective ocular marker for the occurrence of ASI after strabismus surgery. In addition, possible changes in intraocular angiogenic factors during the early stages of ASI have not been identified. The aim of this study was to investigate changes in the aqueous and vitreous levels of PGE2, VEGF, and HIF-1α in an animal model with ASI and to evaluate the effect of TA on ASI.

## Results

A total of 48 eyes in 28 rabbits were included in this study: 20 rabbits with 20 right eyes in the ASI group and 20 left eyes in the contralateral group, and 8 rabbits with 4 right eyes in the control group and 4 right eyes with TA injection in ASI group.

**Figure 1:**
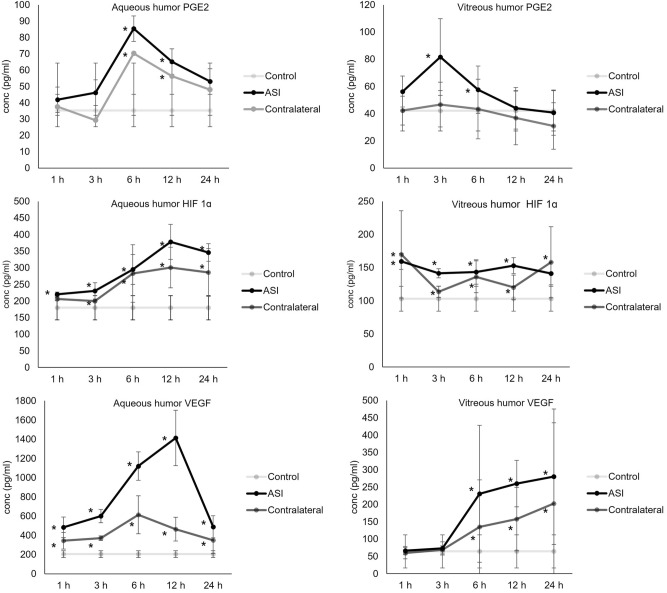
The concentration of angiogenic factors in the aqueous and vitreous humor at each time point. *p < 0.05 compared with control group.

In both the ASI group and contralateral group, the PGE2 concentration in the aqueous humor was elevated from 6 to 12 h, with the peak concentration observed at 6 h (Table 1, Fig. 1). The PGE2 concentration in the vitreous humor was elevated from 6 to 12 h in the ASI group, with the peak concentration observed at 3 h (Table 1, Fig. 1).

**Table 1:** Concentration of prostaglandin E2 in the aqueous and vitreous humor.

Concentration (pg/mL)	Aqueous humor					Vitreous humor				
	Control	ASI	p	Contralateral	p	Control	ASI	p	Contralateral	p
1 h	35.24 ± 11.04	41.82 ± 17.82	0.77	37.53 ± 15.13	0.65	42.09 ± 14.83	56.23 ± 11.41	0.15	42.24 ± 10.63	0.38
3 h		46.20 ± 9.77	0.25	29.32 ± 8.31	0.33		81.65 ± 28.22	0.02	46.67 ± 16.50	0.44
6 h		85.41 ± 21.07	0.02	70.27 ± 16.90	0.02		57.65 ± 17.42	0.04	43.38 ± 21.98	0.25
12 h		65.21 ± 17.88	0.02	56.26 ± 18.67	0.04		44.00 ± 15.29	0.15	36.86 ± 19.79	0.15
24 h		53.02 ± 26.22	0.83	48.02 ± 36.75	0.21		40.74 ± 16.57	0.56	30.91 ± 17.15	0.41

*ASI* anterior segment group, p = Kruskal–Wallis test, compared with control group.

The HIF-1α concentration in the aqueous humor was elevated from 1 to 24 h in the ASI group and 3–24 h in the contralateral group, with both groups showing peak concentration at 12 h (Table 2, Fig. 1). The HIF-1α concentration in the vitreous humor was elevated from 1 to 24 h in both the ASI and contralateral groups.

**Table 2:** Concentration of hypoxia-inducible factor-1 alpha in the aqueous and vitreous humor.

Concentration (pg/mL)	Aqueous humor					Vitreous humor				
	Control	ASI	p	Contralateral	p	Control	ASI	p	Contralateral	p
1 h	180 ± 36.44	220.00 ± 7.12	0.02	206.00 ± 5.16	0.08	103.00 ± 18.71	159.25 ± 11.87	0.02	170.00 ± 65.72	0.02
3 h		230.50 ± 24.89	0.02	200.00 ± 2.83	0.05		141.50 ± 6.76	0.02	113.75 ± 8.50	0.04
6 h		295.00 ± 45.09	0.02	282.67 ± 86.55	0.02		143.25 ± 18.43	0.04	136.17 ± 23.97	0.02
12 h		378.00 ± 52.48	0.02	301.00 ± 61.05	0.02		153.00 ± 11.92	0.02	120.17 ± 18.69	0.02
24 h		346.00 ± 26.68	0.02	286.00 ± 71.68	0.02		141.25 ± 17.13	0.08	158.14 ± 53.74	0.02

*ASI* anterior segment group, p = Kruskal–Wallis test, compared with control group.

In both the ASI and contralateral eye groups, the VEGF concentration in the aqueous humor was elevated from 1 to 24 h, with the peak concentration observed at 12 h in the ASI group and at 6 h in the contralateral group. The VEGF concentration in the vitreous humor was elevated from 1 to 24 h in both the ASI and contralateral groups (Table 3, Fig. 1).

**Table 3:** Concentration of vascular endothelial growth factor in the aqueous and vitreous humor.

Concentration (pg/mL)	Aqueous humor					Vitreous humor				
	Control	ASI	p	Contralateral	p	Control	ASI	p	Contralateral	p
1 h	204.94 ± 35.43	483.97 ± 106.32	0.02	343.97 ± 86.68	0.02	64.58 ± 47.73	66.73 ± 12.08	0.12	59.59 ± 16.67	0.22
3 h		600.79 ± 70.47	0.02	371.17 ± 22.71	0.02		73.03 ± 19.19	0.15	68.71 ± 11.42	0.32
6 h		1120.64 ± 148.63	0.02	614.11 ± 197.97	0.02		230.66 ± 197.46	0.02	134.98 ± 135.78	0.02
12 h		1413.42 ± 288.82	0.02	464.45 ± 123.39	0.02		259.85 ± 67.07	0.02	86.13 ± 91.59	0.02
24 h		488.88 ± 115.26	0.02	348.39 ± 137.56	0.02		280.05 ± 195.52	0.02	202.49 ± 232.99	0.02

*ASI* anterior segment group, p = Kruskal–Wallis test, compared with control group.

**Figure 2:**
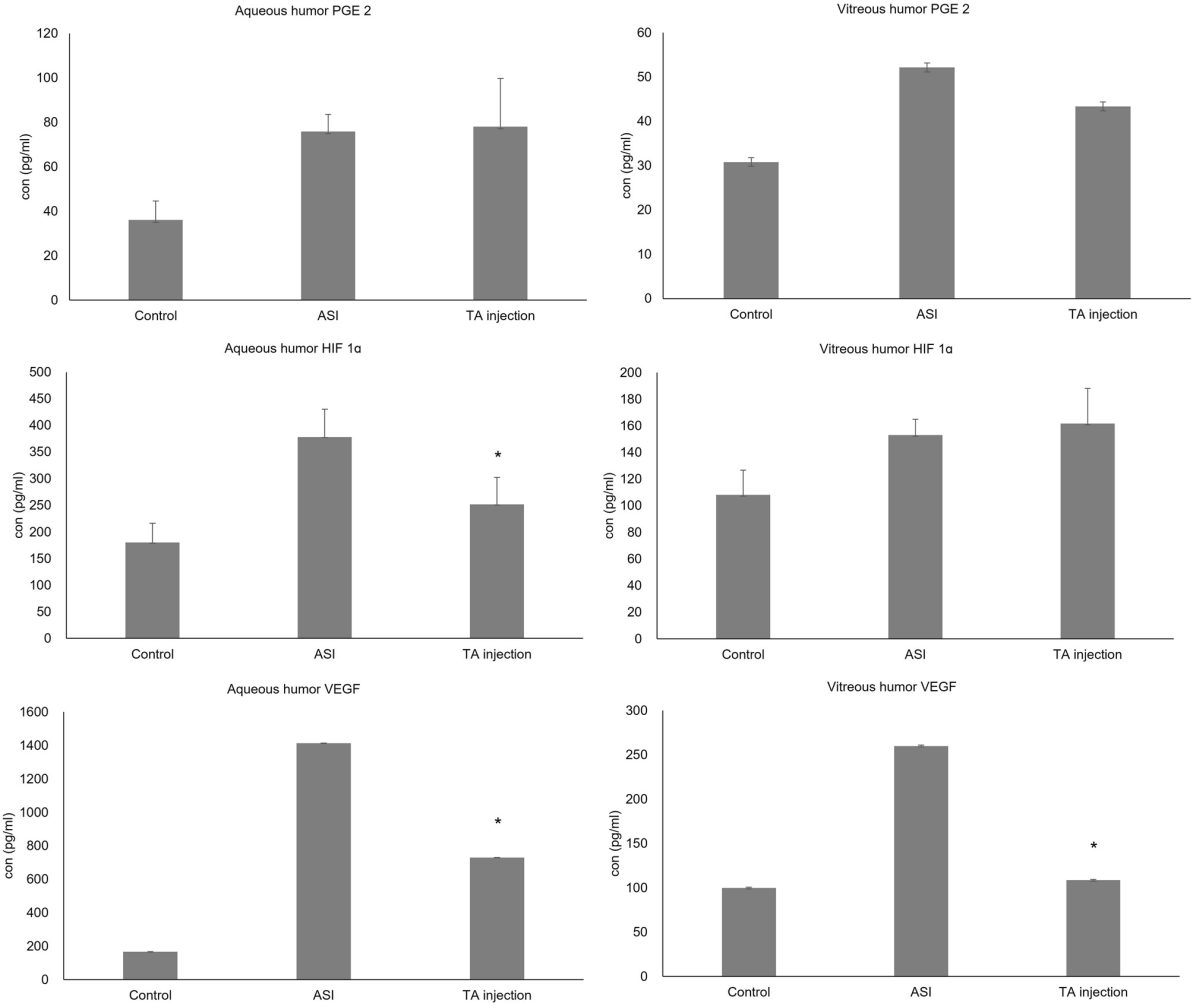
The concentration of angiogenic factors in the aqueous and vitreous humor after triamcinolone acetonide injection *p < 0.05 compared with ASI group.

Figure 2 shows changes in the PGE2, HIF-1α, and VEGF concentrations in the aqueous and vitreous humor with TA injection. There was no significant difference in the PGE2 concentrations in the aqueous and vitreous humor between the TA-injection and ASI groups (p = 0.81, p = 0.59, respectively). The concentration of HIF-1α in the aqueous humor of the TA-injection group was significantly lower than that in the ASI group (p = 0.03); however, there was no significant difference in the vitreous HIF-1α levels between the TA-injection and ASI groups (p = 0.26). The concentration of VEGF in the aqueous and vitreous humor in the TA injection group was significantly lower than that in the ASI group (p = 0.02, all).

## Discussion

This study showed that the concentration of angiogenic factors in the aqueous and vitreous humor changed after disinsertion of the EOM in animals during the initial 24 h. The maximal elevation of the angiogenic factors in the aqueous and vitreous humor was also observed within 24 h. These results indicate that intraocular inflammatory and ischemic reactions occur in ASI within 24 h.

The risk of ASI with an increasing number of operated muscles is well known. Bagheri and colleagues^3^ compared the frequency and severity of ASI after disinsertion of the EOM in a rabbit model. The incidence of ASI was 50% with disinsertion of the four rectus muscles. In their study, ASI was confirmed by ex vivo findings that included the presence of corneal edema, an anterior chamber reaction, or corneal defect; and the eyes with ASI were compared with the fellow eye. In contrast, this study evaluated ASI in the rabbit model by quantitative measurement of intraocular angiogenic factors after disinsertion of the EOM.

The disinsertion of EOM also induced the inflammation in anterior segment part. Ocular inflammation is a pathogenic factor associated with ASI. Intraocular prostaglandin promotes vasodilatation, disrupts the blood–ocular barrier, facilitates leukocyte migration, and interacts with and amplifies many soluble mediators^8^. The corneal angiogenic responses to hypoxic stress have been reported to be initially mediated by cyclooxygenase products of arachidonic acid^9^. As previously reported, PGE2 is involved in the acute phase of ASI and the formation of corneal edema induced by occlusion of the long post-ciliary vessels^10^. In this study, the peak PGE2 concentrations in the aqueous and vitreous humor in the ASI group were observed in the acute phase (6 h and 3 h, respectively). Thus, we suggest that the initial inflammation plays a role in the initiation pathway of ASI.

The synthesis of PGE2 mediates the expression of HIF-1α. HIF-1α is a transcription factor that responds to hypoxia in the cellular environment. HIF-1α in the HIF complex is upregulated in response to hypoxic damage that includes ischemia, trauma, or inflammation^11^. HIF has been speculated to be an intraocular marker for ocular vascular diseases, including diabetic retinopathy or neovascular glaucoma^12,13^. Studies have shown that some of the expression of VEGF is mediated by HIF-1α^14^. In this study, the concentrations of HIF-1α in the aqueous and vitreous humor were elevated in the ASI group within 24 h.

The VEGF is a key stimulator of neovascularization, and its secretion increases during hypoxia. A previous study reported that VEGF plays a role in ASI. Tanaka and colleagues^5^ reported that concentrations of VEGF in the aqueous humor were increased up to 14 d after tenotomy of three EOMs in rabbits. The concentrations of VEGF in the tenotomized eyes were compared with those in the fellow eyes. In our study, the VEGF concentrations in the aqueous and vitreous humor in ASI peaked at 12 and 24 h, respectively.

In previous studies, VEGF is potent stimulator of angiogenesis in hypoxic damage^15,16^. HIF1-α can activate the expression of VEGF. In acute phase of ASI, maximum concentration of HIF1-α and VEGF in aqueous and vitreous humor were observed. Hypoxic cascade including HIF1- α and VEGF signaling might be initiated in 24 h after occurrence of ASI. Measurements of concentration of HIF1-α and VEGF in aqueous and vitreous humor could be intraocular biomarker in ischemic condition.

Additionally, elevated VEGF concentrations in the aqueous and vitreous humor were observed in the contralateral unaffected eye. The amount of increased VEGF concentration was significantly higher in the first 24 h after ASI, compared to that of PGE2 and HIF-1α. This suggests that the VEGF concentration in the aqueous and vitreous humor could be considered as a predictive ocular marker in the early phase of ASI. The concentrations of HIF-1α and VEGF in the aqueous and vitreous humor were also significantly elevated in the unaffected contralateral eyes. The concentration of angiogenic factors in the untreated contralateral eye may be elevated simultaneously through interophthalmic vessels in the rabbit^17^. The unilateral anterior ischemic attack in the rabbit model could affect both eyes by the interocular circulation between the two eyes.

TA inhibits the expression of intraocular neovascularization and inflammation^18,19^. The evaluation of TA injection on PGE2, HIF-1α, and VEGF concentrations in the aqueous and vitreous humor showed that the concentration of HIF-1α in the aqueous humor decreased at 12 h after TA injection. In addition, the concentration of VEGF in the aqueous and vitreous humor decreased significantly after TA injection. Our results indicate that intraoperative TA injection could affect the occurrence of ASI after surgery. VEGF was the only angiogenic factor that was significantly decreased in the aqueous and vitreous humor by TA injection.

This study has several limitations. First, the number of animals used in the study was small. Second, the presence of interocular circulation between the two eyes has not been proven in the human eye. Thus, there is a need to understand the intraocular vascular anatomy from a clinical perspective. Third, the efficacy of only a single concentration of TA was evaluated. Further studies are needed to compare the effects of differing dosages of TA on the angiogenic factors. Lastly, there were anatomical and physiological difference between rabbit and human eye. Rabbit eye has more extraocular muscles including a very strong circular muscle around optic nerve and inserting into the sclera. This variation might affect the concentration of angiogenic factors in this study.

In conclusion, elevation of angiogenic factors in the aqueous and vitreous humor in the eyes with ASI and the contralateral eyes were observed within the initial 24 h following surgery. The peak concentrations of HIF-1α and VEGF in the aqueous humor were observed 12 h after the induction of ASI. Acute unilateral ASI could induce ASI in the fellow normal eye. In addition, the VEGF concentration in the aqueous and vitreous humor decreased 12 h after TA injection. Further experiments will be needed to elucidate the molecular mechanisms of the intraocular ASI pathways.

## Method and materials

### Animals

New Zealand white rabbits weighing 2.0–2.2 kg were used in the study (DooYeol Biotech, Seoul, Korea). The study was carried out in compliance with the ARRIVE guidelines. The procedures adhered to the guidelines of the Association for Research in Vision and Ophthalmology for the Use of Animals in Ophthalmic and Vision Research (ARVO Animal Policy). Approval for this study was obtained from the Institutional Animal Care and Use Committee of Korea University College of Medicine (KOREA-2021–0035).

### Anesthesia

The rabbits were anesthetized with an intramuscular injection of xylazine (0.2 mL/kg Rompun; Bayer Animal Health, Monheim, Nordrhein-Westfalen, Germany) and zolazepam hydrochloride (0.3 mL/kg Zoletil; Virbac, Carros, France). One drop of topical anesthesia with 0.5% proparacaine hydrochloride (Alcaine; Alcon, Fort Worth, TX, USA) was also administered.

### Procedure

All procedures were performed in the right eyes. An incision was made in the conjunctiva 2 mm from the limbus. The four extraocular muscles (inferior, superior nasal, and temporal muscles) were isolated and disinserted from the anterior muscle, including the tendon, from the sclera. The conjunctiva was sutured using an 8–0 polyglactin 910 suture.

### Postoperative management

Topical levofloxacin solution (Cravit eye solution; Santen, Osaka, Japan) was instilled into the postoperative eyes every 2 h.

### Triamcinolone injection

To investigate the efficacy of intralesional TA on ASI in the rabbit model, TA (0.15 mL at 40 mg/mL; Shin Poong Pharm Co., Ltd, Korea) was injected into the subconjunctival space near the detached site at the end of disinsertion of EOM.

### Sample preparation

Four different rabbits were used at each time point for aqueous and vitreous humor collection. The right eyes of rabbits were enucleated and immediately frozen at -80℃. The frozen aqueous and vitreous humor was subsequently separated from the eye. Before analysis, the frozen samples were defrosted and solubilized in 1.0 ml 1% bovine serum albumin (BSA).

### Enzyme linked immunosorbent assay (ELISA)

For the indirect ELISAs to measure concentrations of PGE2 (mybiosource, MBS763445, San Diego, CA, USA), VEGF (R&D Systems, DVE00, Minneapolis, MN, USA), and HIF-1α (mybiosource, MBS2502256, San Diego, CA, USA) in aqueous and vitreous humor, ELISA kits in 96-well plates were used to detect the drug of interest and generate a standard curve of known drug concentrations. The aqueous and vitreous humor samples were divided into aliquots on the plate at 100 µl/well, then Incubated overnight at 4 °C and then washed three times using 200 µl of the washing solution of 0.05% Tween 20 in 1 × phosphate-buffered saline (PBS). The secondary antibody was diluted to 1:20,000 in 0.1% BSA in 1 × PBS. After incubating the plate with a diluted secondary antibody overnight at 4 °C, the optical density was measured at 450 nm wavelength.

### Analysis

The concentrations of PGE2, HIF 1ɑ and VEGF at each time points in ASI, contralateral and control groups were compared. The peaked concentrations of PGE2, HIF 1ɑ and VEGF during initial 24 h in ASI and contralateral group were measured and compared. The change of concentrations of PGE2, HIF 1ɑ and VEGF were measured at 12 h after injection of TA.

### Groups

The animals were divided into four groups: eyes with disinsertion of four rectus muscles (ASI group), contralateral eyes (contralateral group), eyes with TA injection (TA group), and normal eyes (control group). The control group consisted of four healthy normal eyes.

### Statistical analysis

The concentrations between groups were compared using SPSS version 21.0 (SPSS Inc, Chicago, IL, USA). The significance value was set at p < 0.05. Kruskal–Wallis test was used for comparison of concentration between groups.
